# Harnessing the immune microenvironment: advances in nasopharyngeal carcinoma immunotherapy

**DOI:** 10.1038/s41420-026-02999-y

**Published:** 2026-03-10

**Authors:** Yihua Zhu, Yongsheng Liu, Zhikang Yin, Caijing Ou, Xiao’an Guo, Xinru Zhu

**Affiliations:** 1 Jiading Branch of Shanghai General Hospital, Shanghai Jiao Tong University School of Medicine, 800 Huangjiahuayuan Road, Shanghai 201803, People’s Republic of China; 2 Department of Otolaryngology, The First People’s Hospital of Lin’an District, Hangzhou, Zhejiang People’s Republic of China

**Keywords:** Cancer immunotherapy, Cancer microenvironment

## Abstract

Nasopharyngeal carcinoma (NPC), an Epstein–Barr virus (EBV)-driven malignancy with distinct geographic prevalence, presents as locally advanced or metastatic disease in over 70% of patients. Its unique tumor microenvironment (TME) exhibits dense immune infiltration paradoxically coupled with profound immunosuppression orchestrated by EBV through PD-L1 induction, antigen presentation disruption, immunosuppressive cell recruitment, and extracellular vesicle exploitation. These mechanisms underpin the rationale for immunotherapy, where PD-1/PD-L1 inhibitors have transformed management: phase III trials established PD-1 blockade combined with chemotherapy as first-line standard for recurrent/metastatic NPC, significantly improving survival, while integration into locoregionally advanced disease regimens enhances response rates and outcomes. Novel bispecific antibodies and rational combinations (with radiotherapy, anti-angiogenics, and EBV-targeted therapies) show promise in overcoming resistance. Biomarker advances extend beyond PD-L1 to include radiomics, AI-driven models, liquid biopsy markers (EBV-DNA dynamics, exosomal CA1), and tissue-based features (tertiary lymphoid structures, CD70/CD27 axis). Persistent challenges encompass EBV-mediated resistance, biomarker validation, and therapeutic optimization. This review comprehensively synthesizes the mechanistic basis of NPC immune evasion, clinical progress across diverse immunotherapies, biomarker-driven precision strategies, and emerging approaches to harness the immune microenvironment for improved patient outcomes.

## Facts

- EBV-driven immunosuppression underpins NPC pathogenesis: EBV orchestrates immune evasion via PD-L1 exosomal secretion, MHC disruption, and immunosuppressive cell recruitment within the TME, enabling tumor persistence.
- PD-1/PD-L1 inhibitors redefine RM-NPC standard care: ICIs combined with chemotherapy significantly improve survival in recurrent/metastatic disease, with bispecific antibodies showing enhanced efficacy in biomarker-selected subgroups.
- Multimodal synergy optimizes therapeutic outcomes: Radiotherapy-ICI combinations extend PFS in oligometastasis; liquid biopsy and radiomics enable dynamic response monitoring and risk stratification.

## Open questions

- *How to sustainably overcome EBV-mediated resistance?* Can targeting tripartite LMP1-ALIX-PD-L1 complexes or Gal-9/autophagy barriers synergize with cellular therapies to reverse T-cell exhaustion?
- *Which biomarker platform best personalizes immunotherapy?* Can integrated radiomic-TME signatures replace static PD-L1 to predict ICI benefit across disease stages, avoiding spatial/temporal heterogeneity pitfalls?
- *Can toxicity-managed combinations replace chemotherapy backbones?* Will rationally designed ICI/anti-angiogenic/T-cell therapy regimens reduce severe adverse events while maintaining efficacy in geriatric/pediatric populations?

## Introduction

Nasopharyngeal carcinoma (NPC) represents a unique epithelial malignancy originating from the nasopharyngeal mucosa, distinguished by a pronounced geographic predilection [1, 2]. Pathogenesis involves a complex interplay of etiological factors: nearly universal latent Epstein–Barr virus (EBV) infection [3–5], established genetic susceptibility loci contributing to ethnic predisposition [6–9], and significant environmental influences. The anatomical location of the nasopharynx and frequent lack of specific early symptoms result in over 70% of patients presenting with locally advanced or metastatic disease at diagnosis [10–12].

Radical radiotherapy, particularly intensity-modulated radiotherapy, is the cornerstone for non-metastatic NPC [13–16]. For locally advanced disease (LANPC), standard treatment combines induction chemotherapy (IC) with platinum-based concurrent chemoradiotherapy (CCRT), achieving high locoregional control [17–19]. Recurrent/metastatic (R/M) disease relies on systemic therapy, now including PD-1 inhibitors combined with chemotherapy as first-line, alongside anti-angiogenics and targeted agents [14, 20–23]. Despite these strategies, significant challenges lead to poor outcomes. Intrinsic resistance to IC affects about 20% of LANPC patients, increasing distant metastasis risk. Radioresistance contributes to locoregional recurrence. Crucially, distant failure causes over half of NPC deaths. Bone metastases often result in severe, debilitating complications (SREs) that profoundly impair quality of life and predict poor survival [24, 25]. Resistance to therapies and difficult-to-manage treatment toxicities remain major hurdles [26–28]. Understanding the mechanisms driving metastasis and treatment resistance is vital to improve survival.

NPC exhibits a complex tumor immune microenvironment intrinsically linked to its ubiquitous EBV association [9, 29–32]. Virally driven, this malignancy displays dense infiltration of diverse immune cells, notably tumor-infiltrating lymphocytes (TILs) [33, 34]. Paradoxically, this ostensibly immune-rich landscape coexists with potent immunosuppressive mechanisms. NPC tumor cells constitutively express programmed death-ligand 1 (PD-L1) in over 90% of cases [35–37], engaging programmed cell death protein 1 (PD-1) on effector T cells to induce exhaustion and dysfunction [20, 37, 38]. Tumor-derived immunosuppressive factors, particularly interleukin-10 (IL-10) and vascular endothelial growth factor (VEGF), further impair Dendritic cell (DC) maturation and function. Concomitant recruitment and activation of regulatory T cells (Tregs) and myeloid-derived suppressor cells (MDSCs) dampen anti-tumor immunity, fostering a tolerant microenvironment conducive to tumor persistence and progression [39, 40]. Given the complex immunobiology of NPC, characterized by its dense but dysfunctional immune infiltrate and EBV-driven immunosuppression, immunotherapy represents a rational therapeutic strategy [29, 39, 41]. Immune checkpoint inhibitors (ICIs), particularly those targeting the PD-1/PD-L1 pathway, have demonstrated significant clinical benefit and have become an established component of standard care for R/M disease based on landmark phase 3 trials [20, 35, 36]. The application of ICI-based strategies is now also being actively explored and integrated into the treatment paradigm for locoregionally advanced NPC (LANPC). While these advances mark a significant shift in NPC management, overcoming resistance and optimizing patient selection remain key challenges in the field.

This review comprehensively synthesizes current advances in immunotherapy for NPC, focusing on ICIs in R/M and LANPC settings. We evaluate clinical evidence from pivotal trials, analyze mechanisms driving resistance, and assess emerging strategies including EBV-targeted cellular therapies and vaccines. This integrative analysis aims to provide evidence-based guidance for optimizing clinical practice and directing future translational research in NPC immunotherapy.

## Cellular composition and immunosuppressive dynamics of the NPC microenvironment

NPC harbors a profoundly complex and paradoxical tumor microenvironment (TME), fundamentally shaped by its ubiquitous association with EBV. EBV-encoded proteins actively orchestrate this niche, promoting tumor development through interactions with tumor-associated cells while simultaneously limiting immune infiltration of cytotoxic lymphocytes [42, 43]. This milieu features a remarkably dense infiltration of immune cells, predominantly CD8⁺ cytotoxic T cells-a characteristic historically defining NPC as a “lymphoepithelioma” [44–46]. However, this apparent immune richness masks a state of profound dysfunction and active immunosuppression. The TME co-habits both anti-tumor immune effectors and pro-tumorigenic components: Tregs, M2-polarized macrophages, and tumor-supportive B cells promote proliferation by inhibiting CD8⁺ T-cell activity and facilitating metastasis. MDSCs further reinforce immunosuppression, with granulocytic subsets suppressing T cell function via arginase-1-mediated L-arginine depletion and reactive oxygen species (ROS) production [40, 47]. Tumor-associated macrophages often display a hybrid M1/M2 phenotype, paradoxically sustaining inflammation while driving angiogenesis and tissue remodeling [48–50].

Adding structural complexity are tertiary lymphoid structures (TLSs), organized B-cell aggregates associated with favorable prognosis, though their functionality in NPC remains context-dependent [13, 31, 51]. Crucially, stromal components actively fuel malignancy: Cancer-associated fibroblasts (CAFs) secrete a potent secretome that promotes invasion, metastasis [13, 52], and angiogenesis while recruiting/activating immunosuppressive cells. Tumor endothelial cells (TECs) activate survival signaling pathways and produce pro-tumorigenic cytokines, synergizing with CAFs to drive proliferation and dissemination. The extracellular matrix (ECM) serves as a critical cytokine reservoir at both primary and metastatic sites, structurally supporting tumor growth [53, 54].

**Fig. 1:**
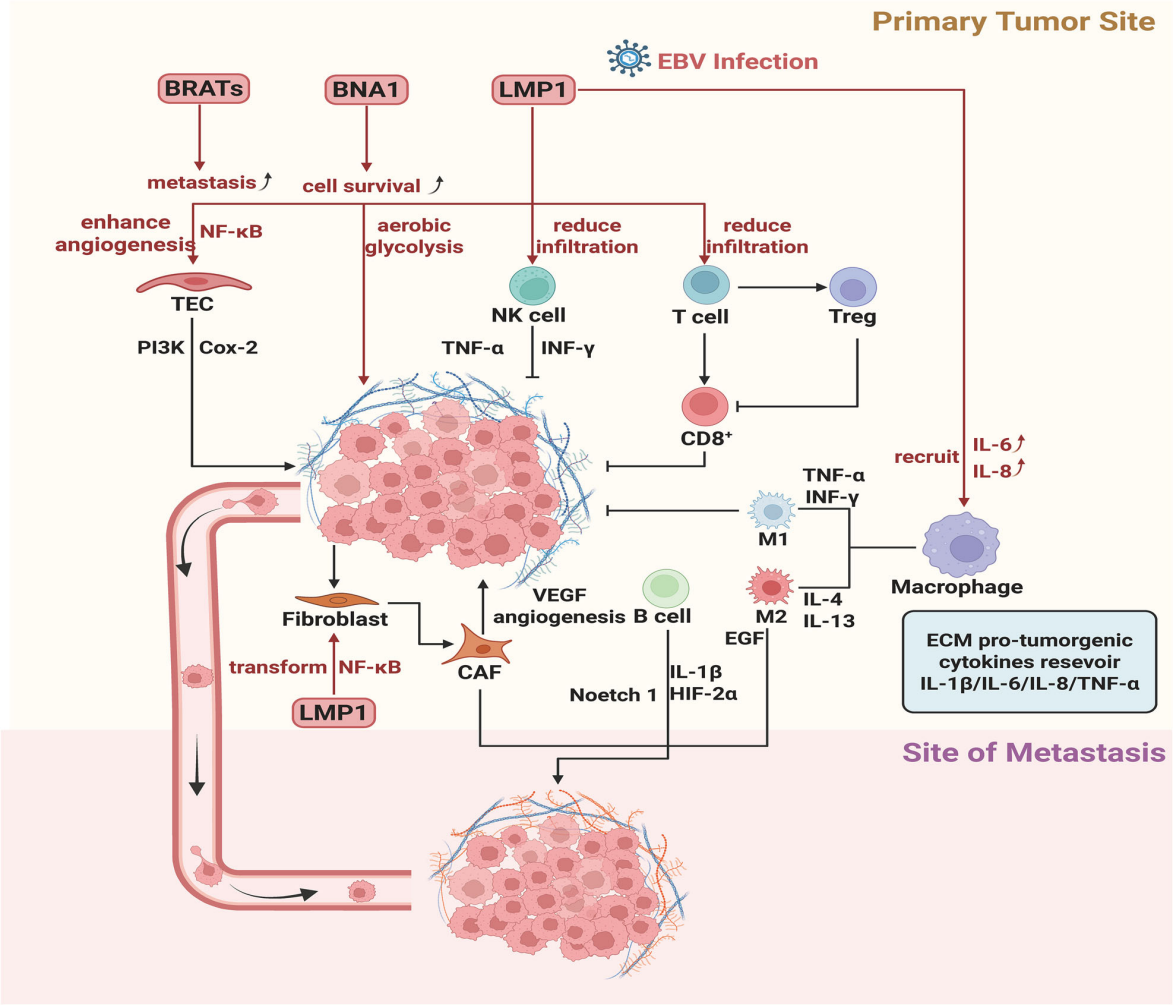
Multifaceted interplay between NPC cells and the tumor microenvironment. EBV-encoded proteins orchestrate tumor progression through stromal reprogramming while constraining T/NK cell infiltration. Antitumor effectors counteract tumor growth, whereas immunosuppressive circuitry promotes metastasis via T-cell inhibition. Stromal components – tumor endothelial cells (TECs) and cancer-associated fibroblasts (CAFs) – activate prosurvival signaling and secrete immunosuppressive factors. The extracellular matrix constitutes a dynamic reservoir of bioactive factors supporting primary and metastatic niche development. *Created with BioRender*.

DCs, essential for immune priming, are functionally crippled within this niche due to tolerogenic LAMP3⁺ DC subsets and tumor-derived soluble factors that suppress maturation, differentiation, and antigen presentation [55]. This culminates in an immunosuppressive landscape characterized by exhausted CD8⁺ T cells expressing inhibitory receptors engaging ligands like PD-L1. Metabolic reprogramming further entrenches immunosuppression: Hypoxia induces IDO1 [56], depleting tryptophan via the kynurenine pathway [57], while nutrient depletion and accumulation of waste products by proliferating tumor/stromal cells collectively inhibit T-cell effector functions [58, 59]. This convergence of suppressive cells, inhibitory receptors, soluble mediators, ECM dynamics, and metabolic stress establishes an immunosuppressive equilibrium that promotes tumor progression (Fig. 1).

## EBV: architect of immune evasion and therapeutic resistance

### Fundamental immune evasion mechanisms

EBV enters the host through oral infection, initially targeting epithelial cells where it activates oncogenic signaling pathways that drive metabolic reprogramming, immune evasion, and genomic instability, ultimately promoting epithelial proliferation and development of EBV-associated carcinomas including NPC [60, 61]. As the principal architect of the NPC immune landscape, EBV establishes type II latency in transformed epithelial cells, expressing latent membrane proteins, Epstein–Barr nuclear antigen 1 (EBNA1), Epstein–Barr Virus-Encoded Small RNA (EBER) RNAs, and BamHI-A Rightward Transcripts (BART) microRNAs (miRNAs) [62–65]. The virus systematically reshapes the TME to evade immune destruction: LMP1, a functional CD40 homolog, constitutively activates key signaling pathways by mimicking Tumor Necrosis Factor Receptor signaling [66, 67], driving pro-survival/metastatic genes while inducing robust PD-L1 expression through direct Nuclear Factor Kappa-Light-Chain-Enhancer of Activated B Cells (NF-κB) binding and indirect Interferon-gamma (IFN-γ) induction [68, 69]. Concurrently, EBV orchestrates dismantling of antigen presentation via downregulation/mutation of MHC class I molecules and aberrant MHC class II expression [70–72]. Viral miR-BARTs provide additional immune regulation by targeting transcripts of antigen presentation, cytokine signaling, and apoptotic genes. Natural Killer (NK) cell surveillance is subverted through EBV-mediated upregulation of inhibitory NK ligands and downregulation of activating ligands [71, 73].

**Fig. 2:**
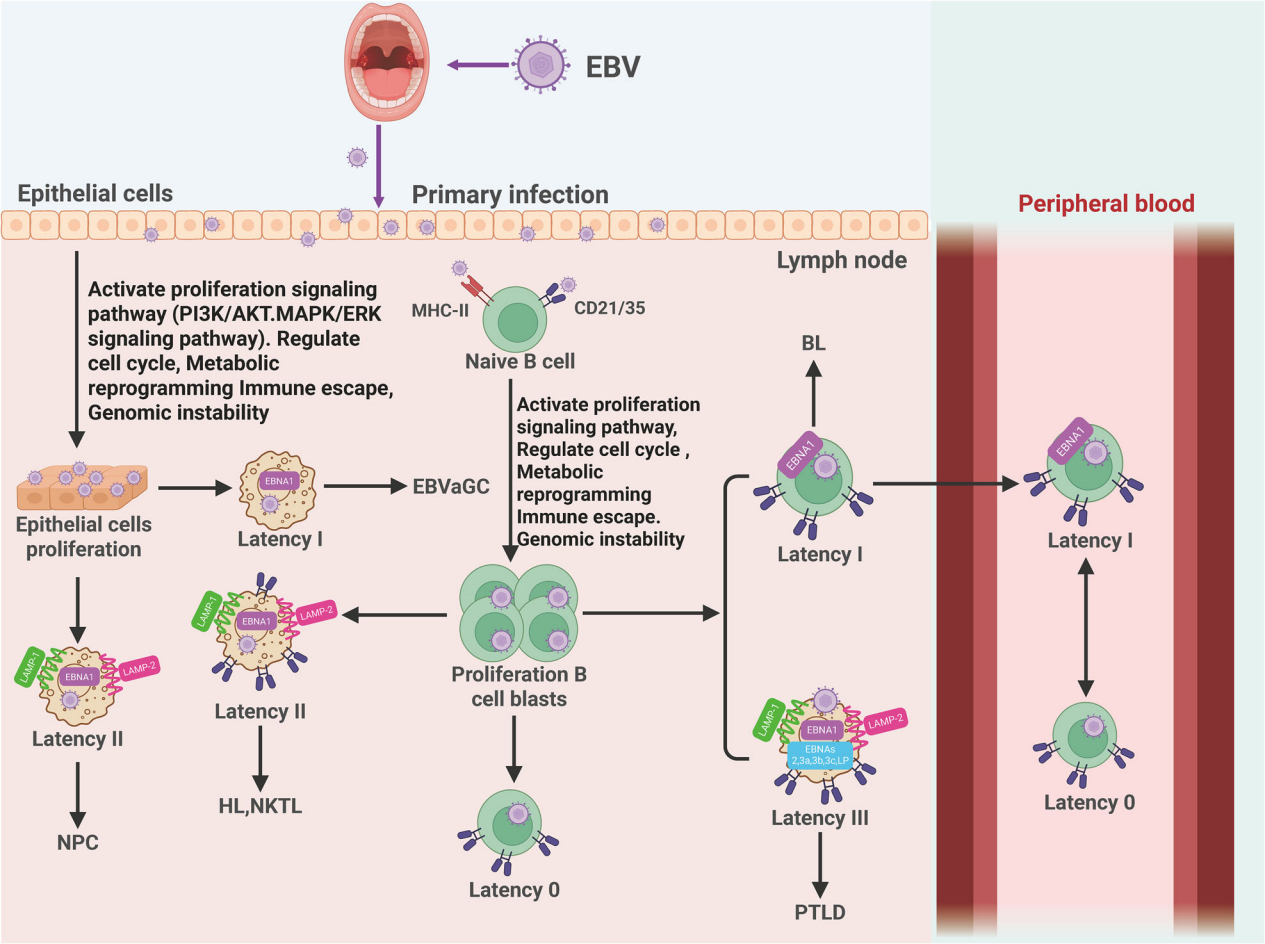
Pathogenic cascade of Epstein–Barr virus (EBV) oncogenesis. EBV initiates epithelial infection via oral transmission, orchestrating PI3K-AKT/MAPK-ERK pathway activation to drive metabolic reprogramming, immunoevasion, and genomic instability, culminating in nasopharyngeal carcinoma (NPC) and EBV-associated gastric carcinoma (EBVaGC) development. Subsequent CD21/35-mediated B-cell infection establishes distinct latency programs: Latency 0 maintains viral genome replication without protein expression; Latency I expresses EBNA1 exclusively; Latency II co-expresses EBNA1/LMP1/LMP2; Latency III exhibits full EBNA/LMP repertoire. Dynamic interconversion between Latency 0 and I states in circulating B lymphocytes facilitates persistent immunoevasion. *Created with BioRender*.

Following epithelial replication, EBV released from infected cells binds CD21/35 receptors on B cells to establish distinct latency programs**:** Latency III, Latency II, Latency I, and quiescent Latency 0 [74, 75]. The interconversion between Latency 0 and I in circulating B cells represents a critical immune evasion mechanism [29, 76]. Meanwhile, EBV masterfully exploits extracellular vesicles (EVs) in epithelial tumors [77–79]; NPC-derived exosomes transport immunosuppressive cargo that systemically modulates immunity [65, 80–83]: Galectin-9 (Gal-9) binds TIM-3 inducing T-cell exhaustion, miR-BARTs reprogram immune cells, exosomal PD-L1 engages PD-1, and DC function is impaired. This comprehensive strategy-combining intrinsic epithelial reprogramming, dynamic B-cell latency switching, and EV-mediated systemic manipulation-neutralizes immune surveillance to foster tumor progression (Fig. 2).

### EBV-mediated therapeutic resistance

**Fig. 3:**
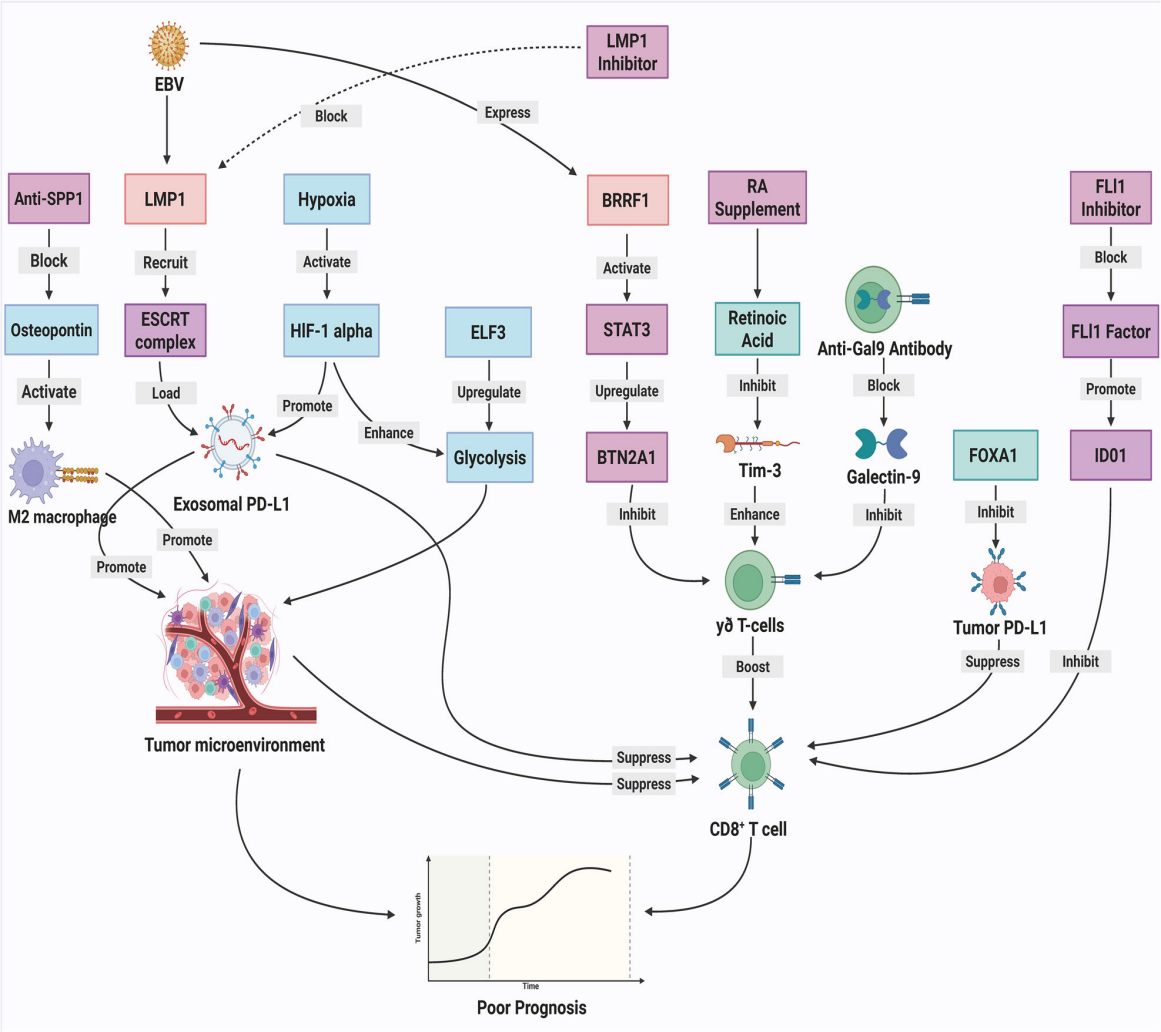
Dynamic immunoregulatory networks in the NPC microenvironment. EBV orchestrates a multifaceted immunosuppressive network by promoting LMP1-ALIX-mediated loading of PD-L1 into exosomes, thereby impairing CD8⁺ T-cell function, inducing BRRF1-driven upregulation of BTN2A1 through JAK3-STAT3 signaling, and facilitating Gal-9-dependent generation of tolerogenic mregDCs. This immunosuppressive milieu is further intensified by hypoxia and stromal reprogramming, as HIF-1α enhances exosomal PD-L1 expression and the SPP1/CD44 axis promotes M2 macrophage polarization, while metabolic disturbances, including ELF3/MUC16-driven glycolytic flux and USP7/TRIM24-mediated M1-to-M2 macrophage switching, further reinforce immune escape. In parallel, several counter-strategies have emerged to reverse these effects, including suppression of the RA/RAR axis to inhibit Tim-3 and alleviate γδ T-cell exhaustion, restoration of FOXA1 to attenuate STAT1-IRF1-PD-L1 hyperactivation, and inhibition of FLI1 to block IDO1-kynurenine-mediated T-cell dysfunction and Treg expansion, collectively highlighting their synergistic therapeutic potential when combined with checkpoint blockade. *Created with BioRender*.

EBV orchestrates multifaceted therapeutic resistance through distinct biological pathways. The LMP1-ALIX complex facilitates PD-L1 loading into exosomes [67], while tumor-derived exosomal Galectin-9 induces mature regulatory dendritic cells that exhibit immunosuppressive properties [84]. EBV-induced CCL5 secretion recruits monocytes polarized into immunosuppressive CD163⁺ M2 macrophages through CSF1 and IL-10 signaling, with resulting MMP9 secretion directly inhibiting T-cell cytotoxicity and promoting exhaustion [85]. Concurrently, tumor cell-surface Galectin-9 binding during cytotoxic lymphocyte contact triggers protective autophagy while suppressing necroptosis, establishing a physical barrier against CTL-mediated killing [58]. These mechanisms are exacerbated by hypoxia-induced HIF-1α activation, which upregulates exosomal PD-L1 expression [86]. Within adoptive cell therapy settings, CD4⁻CD8⁻ double-negative regulatory T cells further reinforce resistance by inhibiting CD8⁺ T-cell expansion through Fas ligand-mediated apoptosis and cytokine secretion [33] (Fig. 3). Key mechanistic insights are systematically categorized in Table 1.

**Table 1:** Mechanisms of therapeutic resistance in nasopharyngeal carcinoma.

Mechanism category	Regulatory target/pathway	Key cellular players	Functional outcome in TME	Reference
Exosomal	LMP1-ALIX complex	Exosomes	Facilitates PD-L1 loading into exosomes	[67]
Exosomal	Tumor-derived exosomal Galectin-9	Mature regulatory dendritic cells	Induces cells that exhibit immunosuppressive properties	[84]
Macrophage polarization	EBV-induced CCL5/CSF1 & IL-10 signaling	Monocytes, CD163⁺ M2 macrophages	Recruits Polarized into M2 macrophages; Resulting MMP9 secretion inhibiting T-cell cytotoxicity	[85]
Autophagy	Tumor cell-surface Galectin-9	Tumor cells, CTLs	Triggers protective autophagy while suppressing necroptosis; Establishing a physical barrier	[58]
Hypoxia	Hypoxia-induced HIF-1α activation	Exosomes	Upregulates exosomal PD-L1 expression	[86]
T-cell suppression	Fas ligand-mediated apoptosis / Cytokine secretion	CD4⁻CD8⁻ double-negative regulatory T cells (DN Tregs), CD8⁺ T cells	Reinforce resistance by inhibiting CD8⁺ T-cell expansion	[33]

## Immunotherapy for NPC: consolidated clinical framework

### ICI clinical research in NPC

ICIs demonstrate significant clinical activity in recurrent/metastatic nasopharyngeal carcinoma (RM-NPC). A meta-analysis of PD-1 inhibitors for platinum-refractory RM-NPC revealed an objective response rate (ORR) of 24% and disease control rate (DCR) of 52%, with encouraging 1-year progression-free survival (PFS) and overall survival (OS) rates of 25% and 53%, respectively[87]. Importantly, PD-L1 expression serves as a predictive biomarker, with PD-L1-positive patients achieving a higher ORR of 31% compared to 21% in PD-L1-negative patients. The safety profile is manageable, with grade 3 or higher treatment-related adverse events (TRAEs) occurring in 19% of patients.

Novel bispecific antibodies provide promising therapeutic alternatives. Cadonilimab, a PD-1/ Cytotoxic T-Lymphocyte-Associated Protein 4 (CTLA-4) bispecific antibody, achieves an ORR of 26.1% in pre-treated RM-NPC patients, with augmented responses noted in individuals exhibiting PD-L1 expression levels of 50% or greater and low EBV-DNA levels [88]. This regimen demonstrates a manageable toxicity profile, with only 8.3% experiencing grade 3 or higher TRAEs, alongside a median PFS of 3.71 months and a 12-month OS rate of 79.7%. Another bispecific antibody, IBI318, shows an overall ORR of 15.5% in advanced cancers in its Phase Ia/Ib trial, rising to 45.5% in treatment-naïve non-small cell lung cancer and 30% in chemo/immunotherapy-naïve NPC patients [89]. Its recommended Phase II dose is 300 mg every 2 weeks, with TRAEs occurring in 85.4% of patients.

For patients progressing after prior ICI therapy, Cadonilimab combined with TPC chemotherapy demonstrates substantial efficacy, achieving an ORR of 68% in this heavily pre-treated population [90]. This combination yields a median duration of response of 9.1 months and median PFS of 10.6 months. While 48% of patients experienced grade 3–4 TRAEs and 56% had immune-related adverse events (irAEs), the regimen represents a valuable salvage option. Importantly, ICIs remain effective and safe for elderly patients, with comparable survival outcomes and toxicity profiles across age subgroups and different ICI agents [91]. The addition of local therapy (LT) to immunotherapy further extends PFS in this demographic.

The optimal application of ICIs is increasingly guided by biomarkers. PD-L1 expression exhibits context-dependent predictive value: while first-line ICIs improve PFS regardless of PD-L1 status, PD-L1 expression 1% or higher correlates with significantly higher ORR in *later-line* settings [92]. Beyond PD-L1, emerging clinical biomarkers include irAEs. Patients developing irAEs, particularly endocrine irAEs, experience superior outcomes including higher ORR, higher DCR, and significantly prolonged PFS and OS following anti-PD-L1 therapy [93]. Conversely, patients experiencing grade 3 or higher irAEs or requiring systemic corticosteroids have shorter OS. The Systemic Immune-Inflammation Index (SII) also demonstrates prognostic utility, as low SII correlates with higher ORR/DCR and significantly longer PFS/OS in RM-NPC patients treated with PD-L1 inhibitors [94].

These findings collectively underscore the expanding role and nuanced application of ICIs across various lines of therapy in RM-NPC. The key efficacy and safety outcomes for monotherapy and combination regimens, alongside the predictive value of major biomarkers, are summarized in Table 2.

**Table 2:** Clinical evidence for immune checkpoint inhibitors in nasopharyngeal carcinoma: monotherapy, combination efficacy, and biomarker-directed therapy.

Study type	Treatment regimen	Patient population	Key efficacy outcomes	Safety profile	Conclusions	References
ICI monotherapy/Combination efficacy						
Meta-analysis	PD-1 inhibitors	Platinum-refractory R/M-NPC	ORR: 24%	Gr≥3 TRAEs: 19%	Safe and efficacious salvage option for platinum-pretreated R/M-NPC	[87]
			DCR: 52%			
			1-yr PFS: 25%			
			1-yr OS: 53%			
			PD-L1 + ORR: 31% vs PD-L1-: 21%			
Phase II trial	Cadonilimab (PD-1/CTLA-4 bispecific)	Previously treated R/M-NPC	ORR: 26.1%	Gr≥3 TRAEs: 8.3%	Effective salvage therapy with manageable toxicity	[88]
			ORR in PD-L1 ≥ 50%/low EBV DNA	Common: Hypothyroidism, rash, pruritus		
			mPFS: 3.71mo			
			12-mo OS: 79.7%			
Phase Ia/Ib trial	IBI318 (PD-1/PD-L1 bispecific)	Advanced solid tumors (ICI-naive NPC subgroup)	Overall ORR: 15.5%	TRAEs: 85.4%	Promising activity in NSCLC and NPC with favorable safety	[89]
			NPC ORR: 30% (platinum/ICI-naive)	Gr≥3 TRAEs: 9.7%		
Phase II study	Cadonilimab + TPC chemotherapy	Immunotherapy-progressed R/M-NPC	ORR: 68% (3 CR, 14 PR)	Gr3-4 TRAEs: 48%	Valuable option for immunotherapy-refractory disease	[90]
			mDoR: 9.1mo	irAEs: 56%		
			mPFS: 10.6mo			
Retrospective study	Various ICIs ± local therapies	Elderly R/M-NPC (≥65 years)	PFS with ICI + local therapy	Safe profile in geriatric population	Safe and effective in elderly; Comprehensive geriatric assessment recommended	[91]
			No survival/toxicity differences across age subgroups or ICI types			
Biomarker-guided therapy						
Meta-analysis	Various ICIs	R/M-NPC (*n* = 1312)	1L: Improved PFS regardless of PD-L1	Not reported	PD-L1 ≥ 1% predicts better ORR in later-line settings	[92]
			2L: ORR with PD-L1 ≥ 1%			
			No benefit at 10%/25% PD-L1 cutoffs			
Retrospective study	Anti-PD-L1 therapy	R/M-NPC	With irAEs vs without:	Gr≥3 irAEs/steroid-requiring: OS	irAEs (especially endocrine) predict enhanced anti-PD-L1 efficacy and survival	[93]
			ORR: 31.2% vs 17.1%			
			DCR: 66.7% vs 44.8%			
			mPFS: 129d vs 56d			
			Endocrine-irAEs:			
			DCR (71.8% vs 46.2%)			
			OS (746d vs 438d)			
Biomarker analysis	PD-L1 inhibitors	R/M-NPC from clinical trials (*n* = 153)	ORR/DCR in low SII	Not reported	SII predicts efficacy and prognosis for PD-L1 inhibitors	[94]
			High SII independently associated with PFS/OS			

### Optimization of combination strategies

The therapeutic synergy between radiotherapy and immunotherapy demonstrates context-dependent efficacy in NPC. For de novo metastatic NPC (dmNPC), combining immunotherapy with locoregional radiotherapy (LRRT) significantly improves PFS and OS compared to immunotherapy alone [95], particularly in oligometastatic disease (OMD), patients with low EBV DNA, and chemoimmunotherapy responders [96]. Patient selection is guided by metastasis burden, EBV DNA status, and treatment response. In locally advanced NPC (LA-NPC), neoadjuvant PD-1 inhibitors with IC substantially enhance complete response (CR) rates. Tislelizumab-based induction achieves 37.2% CR and 93.0% 3-year PFS [97], while camrelizumab-docetaxel-cisplatin induction in non-endemic regions yields 92.8% ORR and 84% 3-year DFS [98].

Dual targeting approaches show significant promise in R/M settings. The VEGFR-2 inhibitor apatinib combined with camrelizumab achieves 38.5% ORR and 6-month median PFS in recurrent/metastatic NPC (R/M-NPC) [99]. Mechanistically, APLNR agonism suppresses PD-L1 expression via blockade of IFN-γ-mediated JAK1/STAT1 signaling while enhancing CD8^+^ T-cell infiltration, creating synergistic antitumor effects when combined with PD-L1 blockade [100]. Similarly, the anti-LAG-3 antibody LBL-007 plus toripalimab yields 33.3% ORR and 10.8-month median PFS, with enhanced responses in LAG-3≥2^+^ tumors [101]. Immunomodulatory mechanisms of high-dose radiotherapy include induction of immunogenic cell death, DC activation, and tumor microenvironment reprogramming [102], while exosomal PTEN dynamics correlate with M2 macrophage polarization and CD8^+^ T-cell activity as predictive biomarkers [103].

Maintenance and local interventions are critical for durable control. In dmNPC patients achieving disease control after chemoimmunotherapy-radiotherapy, extended maintenance immunotherapy improves 2-year PFS to 69.7%, particularly in those with low CD3^+^ T-cell, CD20^+^ B-cell, or PD-L1^+^ tumor infiltration [104]. Combining maintenance immunotherapy with capecitabine further extends PFS in patients with multiple metastases or detectable post-induction EBV DNA [105]. For locoregional management, endoscopic nasopharyngectomy (ENPG) with adjuvant PD-1 inhibition improves 2-year PFS in recurrent NPC without increasing surgical morbidity [106], while adjuvant tislelizumab post-ENPG achieves 94% 1-year PFS [107]. Selected dmNPC patients may achieve durable CRs with chemoimmunotherapy alone [108], though adding metastasis-directed SBRT to camrelizumab failed to improve systemic ORR despite OS prolongation in patients with over 3 lesions [109].

The diverse array of combined therapeutic strategies underscores the complexity and promise of multimodal approaches in NPC. The efficacy outcomes, key patient selection criteria, and salient findings across clinical scenarios are consolidated in Table 3.

**Table 3:** Comprehensive analysis of combined therapeutic strategies in nasopharyngeal carcinoma.

Therapeutic strategy	Study type	Treatment regimen	Patient population	Key efficacy outcomes	Safety Profile	Conclusions	References
Radiotherapy-immunotherapy combinations							
	Retrospective cohort	Chemoimmunotherapy + LRRT	Elderly R/M-NPC (≥65y)	Extended PFS with local therapy + ICI	Safe in geriatric population	Local therapy enhances ICI efficacy in elderly	[91]
	Comparative study	Chemoimmunotherapy + LRRT	De novo metastatic NPC	1-yr PFS/OS vs chemoimmunotherapy alone	Manageable toxicity	LRRT improves survival; Predictive scoring model enables personalized selection	[95]
				Oligometastatic/low EBV DNA subgroups benefit most	Gr3-4 anemia/radiation toxicities		
	Randomized Phase II	Camrelizumab ± SBRT	R/M-NPC	No ORR difference (primary endpoint)	Comparable safety	SBRT adds OS benefit in high metastatic burden disease	[109]
				>3 metastases: OS (exploratory)			
	Retrospective cohort	Chemo-IO ± LRRT	De novo metastatic NPC	3-yr PFS: 53.2% vs 31.2%	Not independently prognostic	LRRT benefits oligometastatic/EBV DNA(-) subgroups	[96]
				Benefit limited to oligometastatic/EBV DNA(-) subgroups			
	Mechanistic study	High-dose RT (≥2Gy)	NPC cell lines	Induces immunogenic cell death	N/A	Radiation primes DC activation for IO synergy	[102]
				Pro-inflammatory cytokines			
				Enhances DC immunogenicity			
	Translational study	RT + Immunotherapy	NPC patients	Exosomal PTEN predicts response	N/A	PTEN as predictive biomarker for radio-immunotherapy	[103]
				Correlates with M2 polarization/CD8+ activity			
	Hypoxia mechanism study	N/A	NPC microenvironment	Hypoxia exosomal PD-L1 via HIF-1α	N/A	Targeting PD-L1+ macrophages may enhance ICI efficacy	[86]
				PD-L1+ macrophages induce CD8+ exhaustion			
Neoadjuvant/Adjuvant immunotherapy (7 studies)							
	Retrospective	IC + anti-PD-1 vs IC	LA-NPC	Induction ORR with IO	Comparable Gr3-4 AEs	Short-term tumor response improvement; Long-term benefit uncertain	[137]
				No difference in post-RT ORR/CR			
	Retrospective	IC + PD-1 inhibitor	LA-NPC	CR: 31.3% vs 4.7%	Hematologic toxicity dominant	Promising neoadjuvant approach; Optimizes tumor debulking	[138]
				1-yr LRFS/DMFS/DFS/OS ≥ 96.9%	No Gr5 events		
	Retrospective PSM	Tislelizumab + chemo → CCRT	LA-NPC	CR: 37.2% vs 12.8%	Well-tolerated	Superior efficacy over standard therapy	[97]
				3-yr PFS: 93.0% vs 78.7%			
				3-yr OS: 95.3% vs 87.2%			
	Clinical study	Chemo-IO vs chemo induction	Stage IV NPC	ORR (95.7% vs 77.8%)	No significant AE difference	Effective and safe for advanced NPC	[139]
				CR (39.1% vs 22.2%)			
				24-mo EFS (88.9% vs 62.6%)			
	Prospective Phase II	Camrelizumab + chemo → CCRT → maintenance	Non-endemic LA-NPC (III-IVA)	3-yr DFS 84%	Gr3-4 leukopenia/neutropenia/weight loss/lymphopenia	Promising activity in non-endemic regions	[98]
				ORR 92.8% (induction), 100% (post-CCRT)			
	Phase II Multicenter	Nivolumab + chemo → RT	NPC	3-yr FFS 88.5%, OS 97.9%	Low toxicity (Gr3-4 acute 40.2%, late 5.2%)	Allows omission of concurrent cisplatin	[140]
				Early EBV DNA clearance FFS			
	Phase II	Camrelizumab + mTPF	LA-NPC	CR 41.4%	Gr3-4 chemo-AEs 26.6%	Highly active and tolerable	[141]
				ORR 100%	irAEs 66.7% (mostly Gr1-2)		
Targeted-immunotherapy combinations							
	Phase II Trial	Apatinib + Camrelizumab	R/M-NPC (*n* = 26)	ORR 38.5%	Tolerable, manageable toxicity	Promising new option	[99]
				mPFS 6 mo			
	Mechanistic Study	APLNR targeting + PD-L1 Ab	NPC models	APLNR PD-L1 expression	N/A	APLNR as potential IO target	[100]
				CD8+T-cell infiltration			
				Tumor suppression vs monotherapy			
	Phase II Study	PD-1 + Nimotuzumab + Chemo	Locoregionally advanced NPC	3-yr PFS: 96.6% vs 79.8%	Gr≥3 AEs 5.3%	Highly active and safe	[142]
				HR 0.16	Gr≥3 irAEs 4.0%		
				ORR 100% vs 99%			
	Ib/II Trial	LBL-007 + Toripalimab	ICI-naive NPC	ORR 33.3%	Manageable safety, no DLTs	Promising activity in ICI-naive patients	[101]
				DCR 75%			
				mPFS 10.8 mo			
				LAG-3≥2+: ORR 28.0% vs 7.7%			
Maintenance therapy optimization							
	Multicenter Retrospective	Maintenance IO	dmNPC after disease control	15-mo MI 2-yr PFS: 69.7% vs 53.5%	Not reported (requires optimization to minimize toxicity)	MI duration should be tailored to immune context	[104]
				Maximal benefit in low immune infiltration			
	Retrospective	Immu/Cape vs Immu	dmNPC after 1L chemo-IO	PFS with combination	Not specified	Capecitabine adds benefit in high-burden disease	[105]
				Benefit in multi-metastatic/EBV DNA(+)			
Local therapy combinations							
	Retrospective PSM	ENPG ± post-op anti-PD-1	Recurrent NPC	2-yr PFS with IO	Controllable surgical/immune AEs	Postoperative immunotherapy improves tumor control	[106]
				2-yr OS comparable			
	Case Report	Chemoimmunotherapy	De novo metastatic NPC (*n* = 2)	Sustained CR > 2 years	Well-tolerated, no severe AEs	LRRT omission feasible in selected patients	[108]
				Good QoL			
	Randomized Trial	Endoscopic surgery + Tislelizumab	Resectable recurrent NPC (*n* = 42)	1-yr PFS: 94% vs 57%	Gr≥3 irAEs 9%	Adjuvant immunotherapy reduces recurrence	[107]
				PFI: 100% vs 60%			

### Novel therapeutic development

Cellular and gene therapies exhibit considerable potential for harnessing EBV-specific immunity against NPC. Preclinical studies demonstrate that mRNA vaccines encoding truncated EBV latent antigens outperform their full-length counterparts [110]. Recombinant LMP2A vaccines incorporating epitope-enriched regions enhance antigen uptake by antigen-presenting cells and synergize with ICIs to suppress tumor growth [111]. A multi-epitope EBV mRNA vaccine combined with ex vivo-expanded NK cells results in synergistic tumor eradication in humanized mouse models [112]. In adoptive T-cell therapy, an optimized rapid expansion protocol for EBV-specific cytotoxic T lymphocytes enables generation of high-purity CTLs within 9 days. However, the phase III VANCE trial revealed the addition of autologous EBV-CTLs to chemotherapy failed to improve OS in R/M-NPC [60].

**Fig. 4:**
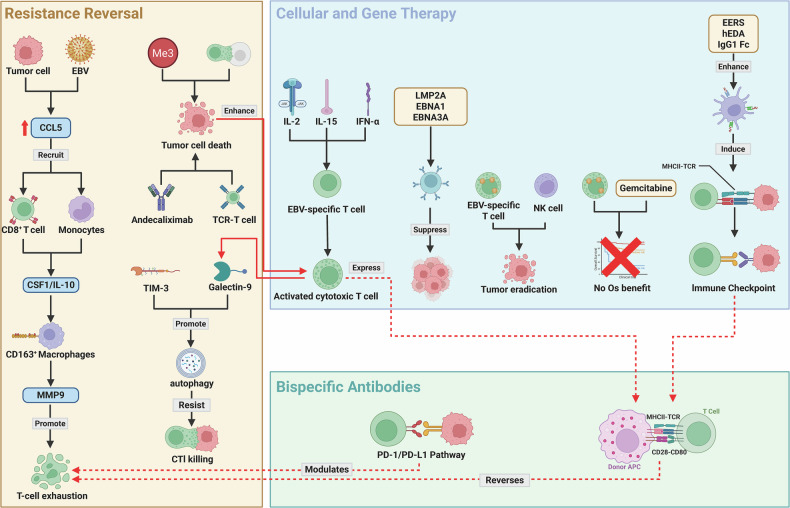
Integrated therapeutic modalities for EBV-driven nasopharyngeal carcinoma. Novel EBV-targeted strategies engage multiple immunologic axes: Truncated antigen mRNA vaccines activate cellular/humoral immunity and synergize with adoptive NK cell therapy for tumor control. Optimized EBV-CTLs expanded via IL-2/IL-15/IFN-α demonstrate enhanced cytotoxicity, though clinical translation requires refinement per phase III VANCE trial findings. Bispecific antibodies targeting PD-1/CTLA-4 (Cadonilimab) and PD-1/PD-L1 (IBI318) reinvigorate T-cell function, with response biomarkers including PD-L1 and EBV-DNA. Resistance mechanisms are concurrently addressed through dual pathways: EBV-induced CCL5 recruits immunosuppressive monocytes that polarize into MMP9-secreting M2 macrophages to promote T-cell exhaustion, overcome by MMP9 inhibition combined with TCR-T therapy; separately, Gal-9-mediated autophagy establishes a cytoprotective barrier against CTL killing, reversible through Gal-9 blockade or autophagy inhibition. *Created with BioRender*.

Bispecific antibodies provide dual-targeting strategies to enhance antitumor immunity. Cadonilimab achieves an ORR of 26.1% in heavily pre-treated R/M-NPC patients [88]. Similarly, IBI318 demonstrated an overall ORR of 15.5% in its Phase Ia/Ib trial. Resistance reversal strategies include pharmacological modulation of the RA/RAR axis to reverse γδ T-cell exhaustion by suppressing Tim-3 expression [113], FLI1-IDO1 inhibition to block kynurenine-mediated Treg differentiation [114], and combinatorial MMP9 inhibitor therapy with TCR-T cells to disrupt EBV-induced resistance networks [85]. FOXA1 overexpression sensitizes tumors to immunotherapy via STAT1-IRF1 axis regulation [115], while autophagy inhibition restores susceptibility to CTL-mediated killing by targeting Galectin-9-induced barriers [58]. These findings are synthesized in Table 4 while the core biological mechanisms underpinning these approaches are illustrated in Fig. 4.

**Table 4:** Novel therapeutic development in nasopharyngeal carcinoma: comprehensive evidence synthesis.

Therapeutic platform	Study type	Mechanism/Target	Key findings	Safety/Toxicity	References
Cellular & gene therapy					
mRNA vaccine	Preclinical (Mice)	Truncated EBV latent antigens (LMP2A, EBNA1, EBNA3A)	Activates cellular/humoral immunity	Not assessed	[110]
			Suppresses tumor progression		
			Extends survival		
			Truncated antigens more effective than full-length		
EBV-CTL production	In vitro Optimization	IL-2/IL-15/IFN-α-enhanced CTL expansion	9-day rapid production without feeder cells	Protocol optimization	[143]
			Yield & cytotoxicity vs IL-2/IL-15 alone		
			Applicable to CMV-CTLs		
EBV-CTLs + Chemo	Phase III RCT (VANCE)	Autologous EBV-CTLs + Gemcitabine/Carboplatin	No OS benefit vs chemo alone	Manageable safety	[60]
			Largest passive T-cell trial in solid tumors (*n* = 330)		
			Safe combination		
Recombinant vaccine	Preclinical	LMP2A EERs + TLR4 agonist + IgG1 Fc	APC antigen uptake	Enhanced immunogenicity	[111]
			MHC-restricted T-cell responses		
			Superior immunogenicity & tumor suppression		
			ICI synergy improves survival		
mRNA vaccine + NK	Humanized Mouse Model	Multi-epitope EBV vaccine + NK cells	Suboptimal efficacy alone	Optimized NK expansion	[112]
			NK cell synergy durable tumor eradication		
			Epitopes from EBV mutations with broad HLA coverage		
Bispecific antibodies					
Cadonilimab	Clinical cohort	PD-1 × CTLA-4 bispecific	26.1% ORR in R/M-NPC	8.3% ≥Gr3 TRAEs (hypothyroidism, rash, pruritus)	[88]
			Response with PD-L1 50%/low EBV-DNA		
			3.71mo mPFS, 79.7% 12-mo OS		
IBI318	Phase Ia/Ib Trial	PD-1 × PD-L1 bispecific	15.5% ORR overall	85.4% TRAEs (≥Gr3: 9.7%)	[89]
			30% ORR in chemo/IO-naïve NPC		
			RP2D: 300 mg Q2W		
Resistance reversal					
MMP9 inhibition	Preclinical (EBV+ models)	Targeting M2 macrophages/MMP9	EBV CCL5 M2 polarization MMP9 secretion	Identifies resistance mechanism	[85]
			MMP9 suppresses T-cell killing		
			MMP9i + TCR-T tumor growth inhibition		
Galectin-9 targeting	In vitro/In vivo NPC	G9-induced autophagy	G9 overexpression autophagy limits necroptosis	Reveals resistance axis	[58]
			Creates anti-CTL defense barrier		
			G9 or autophagy inhibition CTL-mediated killing		
Resistance reversal	Not assessed	RA/RAR axis; Tim-3; γδ T-cell exhaustion	Pharmacological modulation of RA/RAR axis reverses γδ T-cell exhaustion by suppressing Tim-3 expression	Not assessed	[113]
Resistance reversal	Not assessed	FLI1-IDO1 (inhibition); kynurenine-mediated Treg differentiation	FLI1-IDO1 inhibition blocks kynurenine-mediated Treg differentiation	Not assessed	[114]
Resistance reversal	Not assessed	MMP9 inhibitor; TCR-T cells; EBV-induced resistance networks	Combinatorial MMP9 inhibitor therapy with TCR-T cells disrupts EBV-induced resistance networks	Not assessed	[85]
Resistance reversal	Not assessed	FOXA1 (overexpression); STAT1-IRF1 axis	FOXA1 overexpression sensitizes tumors to immunotherapy	Not assessed	[115]
Resistance reversal	Not assessed	Autophagy (inhibition); Galectin-9-induced barriers	Autophagy inhibition restores susceptibility to CTL-mediated killing	Not assessed	[58]

### Biomarkers and precision management

Radiomics biomarkers hold significant promise for non-invasive prediction of treatment response. Deep learning integration with MRI radiomic features achieves high accuracy in predicting clinical complete response to PD-1 inhibitors [116], with MRI-based signatures surpassing PD-L1 expression in predictive value [117]. Further extending these approaches, the GNPC multimodal deep-learning risk score integrates H&E-stained pathology slides and clinical data through graph-based representation of tumor spatial heterogeneity, validated in 1,949 NPC patients to significantly stratify distant metastasis, overall survival, and local recurrence risk, enabling precision treatment beyond TNM staging limitations [118]. Complementing these, a deep learning model (TILDL) quantifying TILs from H&E whole-slide images was developed across 435 nonmetastatic and 63 ICB-treated metastatic NPC patients, demonstrating strong correlations with IHC-derived TIL densities and independent prediction of improved 5-year survival in nonmetastatic cohorts and superior 3-year PFS with immunotherapy in metastatic disease [119]. Similarly, a deep-learning algorithm quantifying 12 TIL density clusters from H&E whole-slide images of 367 NPC patients significantly stratified locoregional recurrence risk, distant metastasis-free survival, PFS, and regional recurrence-free survival, demonstrating independent prognostic value for recurrence beyond conventional TNM/EBV DNA markers [120]. Building on multimodal integration, the CRP prognostic model combining clinical, radiomic (MRI), and pathomic features outperformed comparator models in 268 de novo metastatic NPC patients receiving PD-1 inhibitor plus chemotherapy, achieving C-indexes of 0.823/0.787/0.772 across cohorts. Critically, CRP-stratified low-risk patients showed superior survival with LRRT, enabling personalized sequential therapy selection [121].

Pre-immunotherapy MRI analysis predicts treatment response and OS in LA-NPC. Liquid biopsy biomarkers include sEV-derived CA1 as a superior diagnostic biomarker [122], RA/RAR signaling as a therapeutic target for reversing T-cell exhaustion [113], and plasma-based SERS for monitoring treatment response [123]. Inflammatory and metabolic indicators such as SII, LMR [124], HDL-C [125], LIPI [126], and KMO [127] serve as accessible prognostic tools. Early CRP kinetics and EBV DNA clearance enhance PFS prediction. Tissue biomarkers, including the CD70/CD27 axis [128], CXCL1⁺FAP⁺ CAFs [129], and TLS subtypes [130, 131], and spatial microenvironment architecture—demonstrated in 160 lymphoepithelial carcinomas where semi-supervised H&E segmentation identified prognostically significant lymphocyte gradients and PDL1⁺ tumor cell/FOXP3⁺ Treg spatial arrangements stratifying prognosis.

Advanced prognostic tools include the M1 subclassification stratifying metastatic NPC by bone involvement and lesion count [132]; the RPA-M1 classification incorporating metastasis number and involved organs; LRRT benefit prediction models using serum LDH, EBV DNA status, and metastatic burden [133]; risk-adapted metastasis-directed therapy selection [134]; and PET-CT-based nomograms integrating radiomics and clinicopathological factors. Key predictors and their clinical validation are consolidated in Table 5.

**Table 5:** Biomarkers and prognostic prediction in nasopharyngeal carcinoma: comprehensive evidence synthesis.

Category	Biomarker type/Model	Patient population	Key findings	Prognostic/Predictive value	References
Radiomics biomarkers					
Combined MRI model	Tf_Radiomics+Resnet101	Advanced NPC (PD-1 + chemo)	Predicts clinical complete response (cCR) with high accuracy	Predictive for treatment response	[116]
MRI-based signature	Radiomics vs PD-L1 IHC	NPC (immunotherapy)	Superior predictive value vs PD-L1	Predictive & prognostic	[117]
			Independent predictor of response		
			Radiomics score PFS		
Pre-treatment MRI model	Radiomics/Clinical integration	LANPC (PD-1 inhibitors)	Outperforms clinical models in response prediction	Predictive & prognostic; Biological basis confirmed	[144]
			Comparable to combined model for OS		
			Features correlate with TME pathology		
Liquid biopsy biomarkers					
sEV proteomics	CA1 (Carbonic Anhydrase 1)	NPC (early/late stage)	Diagnostic sensitivity: 81.8-82.7%	Diagnostic biomarker (AUC = 0.89)	[122]
			Superior to EBV-DNA in early NPC		
			Automated chemiluminescence chip developed		
Retinoic acid pathway	Serum RA/Tim-3 on γδ T cells	NPC patients	Serum RA negatively correlates with Tim-3	Predictive for γδ T-cell immunotherapy	[113]
			RA supplementation Tim-3 + anti-tumor immunity		
			Enhances Vδ2 T-cell efficacy		
Plasma SERS	Machine learning model	NPC (pre/post immunotherapy)	High sensitivity (93.3%)/specificity (86.0%)	Monitoring tool for immunotherapy response	[123]
			Avoids “protein corona” interference		
			Distinguishes pre/post-treatment states		
Inflammatory/Metabolic indicators					
SII/LMR dynamics	Systemic Immune-Inflammation Index	NPC (ICI-treated)	Baseline SII + LMR PFS	Predictive & prognostic	[124]
			SII post-treatment + LMR PFS (independent)		
HDL-C metabolism	Serum HDL-C levels	Advanced NPC (immunotherapy)	Baseline HDL-C PFS	Predictive biomarker; Therapeutic implications	[125]
			HDL-C post-treatment PFS (independent)		
			ApoA1 mimetics M2 M1 macrophage repolarization		
LIPI score	Lung Immune Prognostic Index	R/M NPC (ICI-treated)	LIPI OS/PFS/DCR	Prognostic for ICI outcomes	[126]
			Persistent poor LIPI worst outcomes		
Mitochondrial gene	KMO expression	NPC tissues	KMO survival	Prognostic biomarker & therapeutic target	[127]
			Correlates with OXPHOS/glycolysis		
			Associates with B-cell infiltration + inflammatory signatures		
Treatment response dynamics					
CRP kinetics	Early CRP changes	De novo mNPC (chemoimmunotherapy)	CRP responders (after transient) ORR/PFS	Early predictor for treatment response & survival	[145]
			Independent prognostic factor		
			Combined with EBV DNA clearance improves prediction		
Tissue microenvironment biomarkers					
CD70/CD27 axis	Tumor CD70+CD27+ lymphocytes	NPC patients	CD27+ lymphocytes survival	Prognostic; Predicts response to CD70-targeted therapy	[128]
			Tumor CD70 survival (in CD27+ cases)		
			Serum sCD27 reflects tumor interaction		
CAF phenotype	CXCL1+_FAP+ CAFs	NPC patients	High-risk group DMFS/OS/PFS/LRFS	Stratifies metastasis risk; Predicts anti-PD-1 response	[129]
			Independent prognostic factor		
			Associates with PD-L1 expression		
TLS classification	Tertiary Lymphoid Structures	NPC (ICI-treated)	TLS+ (“immune-inflamed”) survival	Prognostic & predictive biomarker	[130]
			T/B cell diversity + immune infiltration		
			Predicts PD-1 blockade response		
IFN-HEV signature	CTRscore (IFN-HEV based)	NPC (ICI-treated)	IFN-HEVs drive TLS formation via CXCL9	Predictive for ICB efficacy	[131]
			Associates with survival + ICB response		
			Predictive model developed		

### Special populations and clinical translation

Elderly patients with RM-NPC derive comparable efficacy and safety benefits from ICIs when augmented with local interventions [91]. Pediatric NPC management utilizes response-adapted, dose-reduced radiotherapy after induction chemoimmunotherapy to minimize late effects [135]. Cost-effectiveness analyses confirm toripalimab-chemotherapy combinations are economically viable [136]. Proactive toxicity management is facilitated by validated nomograms predicting RIOM risk based on neutrophil-to-lymphocyte ratios and induction chemotherapy regimens. Individualized approaches include pembrolizumab-based regimens for bone marrow metastasis and the PRaG regimen for osseous metastases. Key mechanistic insights are systematically categorized in Table 6.

**Table 6:** Mechanisms of Tumor microenvironment (TME) regulation in nasopharyngeal carcinoma.

Mechanism category	Regulatory target/Pathway	Key cellular players	Functional outcome in TME	Therapeutic implication	References
Immunosuppressive mechanisms					
Macrophage polarization	USP7/TRIM24/SPLUNC1 axis	Macrophages (M1/M2)	USP7 stabilizes TRIM24 SPLUNC1 M1 polarization	USP7 overexpression or TRIM24 targeting promotes anti-tumor immunity	[146]
			TRIM24 depletion M2 shift tumor progression		
Glycolytic immune escape	ELF3/MUC16 axis	Tumor cells	Hypomethylated ELF3 MUC16 glycolytic reprogramming	Targeting ELF3/MUC16 restores anti-tumor immunity	[147]
			MUC16 knockdown immune evasion		
M2 macrophage induction	SPP1-CD44/JAK2/STAT3	M2 macrophages	SPP1 M2 polarization via CD44/JAK2/STAT3	SPP1 blockade reverses immunosuppression	[148]
			Correlates with advanced stage/poor prognosis		
EBV immunomodulation	BRRF1/JAK3-STAT3/BTN2A1	Vγ9Vδ2 T cells	BRRF1 BTN2A1 via JAK3-STAT3	Targeting BRRF1 enhances γδ T-cell function	[69]
			IL-22RA2 IL-22 BTN2A1		
			Critical for Vγ9Vδ2 T-cell anti-tumor activity		

## Discussion and prospects

Immunotherapy has fundamentally reshaped the therapeutic landscape for NPC, transitioning from a promising concept to a validated clinical reality. The integration of ICIs, particularly those targeting the PD-1/PD-L1 axis, with standard chemotherapy has established a new survival-prolonging standard of care for R/M disease. This success has extended into the curative-intent setting for LANPC, where incorporating ICIs into induction, concurrent, and adjuvant phases alongside chemoradiotherapy has demonstrably enhanced CR rates and survival outcomes. Beyond conventional ICIs, the development of bispecific antibodies targeting dual checkpoints and the strategic exploration of synergistic combinations-with radiotherapy, exploiting its immunogenic effects, anti-angiogenics countering VEGF-mediated immunosuppression, and targeted agents-represent significant strides in broadening efficacy and overcoming inherent limitations. The synergy between radiotherapy and immunotherapy, particularly in OMD, harnesses radiation-induced antigen release to potentiate systemic immune responses. Concurrently, EBV-directed strategies, including adoptive T-cell therapies and next-generation vaccines, leverage the tumor’s viral etiology for antigen-specific targeting. Advances in biomarker science have propelled the field beyond PD-L1, embracing radiomic signatures predictive of response, liquid biopsy analytes like sEV-derived CA1 and EBV DNA kinetics, dynamic inflammatory indices, and tissue-based immune architectures such as TLS and the CD70/CD27 axis, collectively advancing the goal of precision immunotherapy.

Despite these transformative achievements, formidable challenges persistently define the frontier of NPC immunotherapy. Paramount among these is the pervasive issue of primary and acquired resistance, an intricate defense orchestrated by the EBV within the immunosuppressive TME. EBV employs multifaceted evasion tactics: constitutive PD-L1 expression coupled with exosomal shedding for systemic suppression, deliberate disruption of MHC class I/II antigen presentation, induction of profound T-cell exhaustion via upregulation of co-inhibitory receptors, recruitment and polarization of immunosuppressive cellular networks, and metabolic rewiring fostering hypoxia, IDO1/kynurenine accumulation, and adenosine generation. This sophisticated network renders a substantial patient subset non-responsive or leads to eventual relapse. Current combination strategies, while improving response rates, frequently encounter significant toxicity hurdles, such as nasopharyngeal necrosis with anti-angiogenic agents or synergistic organ-specific irAEs, necessitating refined management protocols and predictive toxicity biomarkers. The identification and validation of robust predictive biomarkers remains a critical unmet need. Despite numerous promising candidates, including spatially resolved PD-L1, TLS density, dynamic EBV DNA load, radiomic scores, and the occurrence of specific irAEs, their utility is hampered by insufficient validation in large prospective cohorts, lack of assay standardization, and the confounding effects of spatial and temporal heterogeneity within the evolving TME under therapeutic pressure. Furthermore, optimizing the sequencing and integration of diverse modalities-chemotherapy, radiotherapy, targeted therapies, various ICI classes, and cellular therapies-demands rigorous clinical investigation to maximize therapeutic synergy while minimizing antagonism and cumulative toxicity. Tailoring approaches for special populations, such as the elderly requiring comprehensive geriatric assessment and children needing strategies to mitigate long-term radiation sequelae through response-adapted dose reduction, is essential. Finally, demonstrating cost-effectiveness and ensuring equitable access to increasingly complex and costly regimens are vital for sustainable real-world implementation and global health equity.

The path forward necessitates a concerted, multi-pronged translational research strategy focused on dismantling resistance networks and personalizing therapy. Deciphering resistance and identifying novel targets requires deep molecular profiling using single-cell and spatial multi-omics across treatment timepoints to map the dynamic evolution of escape mechanisms within distinct TME niches. This will unveil vulnerabilities beyond current checkpoints, such as disrupting CAF signaling pathways and the ECM barrier, targeting immunosuppressive metabolic pathways, and developing strategies to reverse T-cell exhaustion via epigenetic modulators, engineered cytokine therapies, or targeting key exhaustion-associated transcription factors. Countering EBV-specific evasion involves enhancing antigen presentation, inhibiting critical viral proteins, and boosting NK cell function, alongside exploring novel co-inhibitory and co-stimulatory immune receptors. Developing next-generation biomarkers and personalization mandates moving beyond static markers towards integrated, dynamic platforms employing multiplexed spatial profiling to quantify immune-stromal interactions and functional states within the TME, refining liquid biopsy for real-time monitoring of ctDNA, exosomal cargo, and immune signatures, validating radiomic signatures linked to biology in multi-center trials, and developing functional immune assays to test tumor sensitivity ex vivo. Artificial intelligence will be crucial for integrating complex multi-omic, radiomic, and clinical data into predictive algorithms for treatment selection and outcome forecasting.

Optimizing therapeutic integration and managing toxicity necessitates prospective clinical trials to define optimal treatment sequencing and maintenance therapy duration, coupled with designing mechanistically synergistic combinations that prioritize non-overlapping toxicity profiles. This requires developing validated biomarkers and predictive nomograms for severe irAEs, establishing standardized protocols for managing unique toxicities such as cytokine release syndrome, and conducting dedicated trials for special populations-geriatric patients requiring comprehensive assessment for efficacy and dosing optimization, and pediatric cohorts benefiting from advanced radiotherapy techniques to mitigate late effects. Concurrently, advancing EBV-specific immunotherapy entails designing next-generation multi-antigen vaccines employing mRNA/viral vector platforms with potent adjuvants and heterologous prime-boost strategies, developing enhanced off-the-shelf cellular therapies including TME-armored EBV-CTLs and persistence-engineered CAR-T/NK cells, and rigorously testing these modalities in combination with ICIs, TME modulators, or epigenetic agents through well-designed clinical trials. Advancing EBV-specific immunotherapy entails designing next-generation vaccines targeting multiple EBV antigens with potent adjuvants and heterologous prime-boost strategies, developing enhanced off-the-shelf cellular therapies armored against the TME and with improved persistence, and rigorously testing these EBV-directed modalities in combination with ICIs, TME modulators, or epigenetic drugs in well-designed clinical trials.

In conclusion, immunotherapy has irrevocably altered the prognosis and management paradigm for NPC, offering significant survival benefits, especially in advanced stages. The unique EBV-driven immunobiology provides both the rationale and fertile ground for continued innovation. However, overcoming the sophisticated, virally orchestrated immunosuppressive networks remains the central challenge. Future progress hinges on a deeper systems-level understanding of dynamic resistance mechanisms, the development of mechanism-informed, rationally combined therapeutic strategies with improved safety profiles, the implementation of sophisticated biomarker-guided personalization, and dedicated efforts addressing the needs of special populations and healthcare system implementation. By fostering deeper integration of insights from tumor immunology, virology, molecular biology, and clinical research, the next wave of advances holds the potential to transform NPC into a more consistently controllable and potentially curable malignancy, unlocking the dormant anti-tumor immunity within its uniquely complex microenvironment. The focus must now shift from incremental improvements to fundamentally re-engineering the host-tumor interaction towards sustained immune control.

## Conclusion

Immunotherapy has fundamentally reshaped the therapeutic paradigm for NPC, leveraging its unique EBV-driven immunobiology to achieve unprecedented survival gains in R/M and locoregionally advanced disease. Despite transformative success with PD-1/PD-L1 inhibitors and rational combinations, overcoming sophisticated EBV-orchestrated resistance within the immunosuppressive TME remains the pivotal challenge. Future progress hinges on deciphering dynamic immune evasion mechanisms through multi-omics approaches, developing biomarker-guided personalized strategies, and advancing synergistic combinations to unlock durable anti-tumor immunity across all disease stages.
